# Supplementary material for: An Algorithm for Template-Based Prediction of Secondary Structures of Individual RNA Sequences
Source: Front Genet. 2017 Oct 10;8:147. doi: 10.3389/fgene.2017.00147 (PMC5641303; doi:10.3389/fgene.2017.00147)
Supplement: Supplementary file 1 [file Presentation1.PDF]

SUPPLEMENTARY MATERIAL

Table S1. Sequence representation of the copy step of the proposed method using SAM I secondary structure. Template: *T. tengcongensis* SAM I. Query: *Gramella forsetii* SAM I. Notation: base pairs: '(' and ')', unpaired nucleotides: '.', gaps: '-', false non-canonical base pairs: '3', gapped base pairs: '1'.

```
Template sequence:

GGCUUAUCAAGAGAGGUGGAGGGACUGGCCCGAUGAAACCCGGCAACCAGAAAUGGUGCCAAUUCCUGCAGCGGA
AACGUUGAAAGAUGAGCCA

Template structure:

((((((((....(.(((...(((.....)))......))))(((..(((....))).)))...(....(((((..
..)))))..))))))))).

Query sequence:

UCGUUAUCAAGAAAGGUUGAGGGAUUAGACCCUGUGAAACCUUGGCAACCCUCCUGUAAUGGGAGAAGGUGCUAC
GUUCUACCACGCCAGUCGUGGAUAGAUAACUC

Alignment of template and query sequences:

-GGCUUAUCAAGAGAGGUGGAGGGACUGG-CCCGAUGAAACCC-GGCAACC-----------
AGAAAUGGUGCCAAUUCCUGCAGCGGAAA-CGUUGAAAGAUGAGCCA

UCG-
UUAUCAAGAAAGGUUGAGGGAUUAGACCCUGUGAAACCUUGGCAACCCUCCUGUAAUGGGAGAAGGUGCUACGUU
CUACCACGCCAGUCGUGGAUAGAUAACUC-

Intermediate structure (Figure 1C):

-3(-(((((....(.(((...(((.....-)))......))))-(((..((-----------
3....3)).)))...(....(((((....-)))))..)))))))1)3-
```

Table S2. Characteristics of the large-scale evaluation dataset.

RNA	# of sequences	Avg. sequence length (nucleotides)	Avg. pairwise sequence similarity (%)	Avg. pairwise sequence similarity to template (%)
u4 snRNA	142	144.49	58.594	64.972
tRNA Gly	29	68.345	80.576	86.655
oxyS	11	109.55	80.255	85.091
u5	155	117.09	58.279	63.877
FMN riboswitch	120	136.87	66.554	66.675
THF riboswitch	47	99.383	62.736	61.638
SRP bact small	97	97.969	56.142	58.124
SRP Metazoa	45	291.47	86.904	91.8
TPP riboswitch	13	78.846	95.179	95
IRES HCV	34	220.82	79.547	66.735
SAM I	254	113.43	60.914	64.197
Lysine riboswitch	46	183.22	51.903	45.739
6S	149	180.01	48.214	51
spot42	22	116.55	80.286	84
gcvB	16	200.19	66.1	74.5
Bs2	94	110.51	44.796	51.787
ryhB	5	95	66.6	79
ms1	33	284.94	73.098	74.152
Msmest	486	304.33	56.755	58.93
Telomerase RNA	29	457.76	62.966	70.034
u2 snRNA	191	190.14	61.94	69.607
u1	73	161.82	63.525	70.753

5S	38	116	86.984	84.553
RNaseP nuc	88	312.68	51.245	60.045
CPEB3 ribozyme	12	76.833	84.03	89.917
RNaseP arch	55	315	53.499	46.691
Cobalamin riboswitch	245	205.23	52.462	54.082
u6	140	106.99	68.039	60.671
Tetrahymena ribozyme	11	379.27	45.164	42.727
group I ribozyme	12	364.83	43.439	37.917
Mammalian 18S	71	1787.4	89.0	93.3
RNaseP bact a	429	380.78	58.961	59.555

Table S3. Sources of the large-scale evaluation dataset.

RNA	Source of sequences	Source of template structures
u4 snRNA	RF00015 *	*H. sapiens* u4
tRNA Gly		*H. sapiens* tRNA Gly (PDB ID 5E6M)
oxyS	RF00035	*E. coli* oxyS (1)
u5	RF00020	*H. sapiens* u5 (2, 3)
FMN riboswitch	RF00050	PDB ID 3f2y
THF riboswitch	RF01831	PDB ID 4lvv
SRP bact small	RF00169	*E. coli* SRP (SRPDB ID esccol3d-97-11-17-stretched.pdb)
SRP Metazoa	RF00017	*H. sapiens* SRP (SRPDB ID homsap3d-97-11-17.pdb)
TPP riboswitch	RF00059	*E. coli* TPP (PDB ID 4nyg)
IRES HCV	RF00061	*H. sapiens* IRES HCV (PDB ID 5a2q)
SAM I	RF00162	*T. tengcongensis* SAM I (PDB ID 2GIS)
Lysine riboswitch	RF00168	*T. maritima* lysine riboswitch (PDB ID 4erl)
6S	RF00013	*E. coli* 6S (4)
spot42	RF00021	*E. coli* spot42 (5)
gcvB	(6)	*S. typhimurium* gcvB (6)
Bs2	RF00013	*B. subtilis* Bs2 (7)
ryhB	(8)	*E. coli* ryhB (8)
ms1	RF02566	*M. smegmatis* ms1 (9)
Msmest	(10)	*M. smegmatis* ms1 (9)
Telomerase RNA	RF00024	*H. sapiens* Telomerase RNA (11)
u2 snRNA	RF00004	*H. sapiens* u2 (12)
u1	RF00003	*H. sapiens* u1 (12)
5S	RF00001	*E. coli* 5S (PDB ID 1C2X)
RNaseP nuc	RF00009	*H. sapiens* RNaseP (13)
CPEB3 ribozyme	RF00622	*H. sapiens* CPEB3 (14, 15)
RNaseP arch	RF00373	PDB ID 3q1r
Cobalamin riboswitch	RF00174	PDB ID 4gxy
u6	RF00026	PDB ID 5lqw
Tetrahymena ribozyme	RF00028	PDB ID 1x8w
group I ribozyme	RF00028	PDB ID 1y0q
Mammalian 18S	Silva database (16)	*H. sapiens* 18S rRNA (PDB ID 4v6x)
RNaseP bact a	RF00010	*T. tengcongensis* RNaseP bact a (PDB ID 3q1r)

* RFxxxxx stands for Rfam ID.

Table S4. Cross-validation with experimentally identified structures. Sources of experimentally identified structures (either a paper cited or a four-character PDB ID) are shown in parenthesis in the 1st and 2nd columns. FE is shown in Kcal/Mol. Note that the experimentally identified structures of query RNAs, whose sequences are the query sequences, are compared to generated/predicted structures.

Predictions	Experimentally identified structures (for query)	structure generated by proposed method		structure predicted by RNAfold		structure predicted by refold method		structure predicted by Rsearch-based method	
		tree edit distance	correct base pairs (%)	tree edit distance	correct base pairs (%)	tree edit distance	correct base pairs (%)	tree edit distance	correct base pairs (%)
6S E.c.(6S B.s.)	(4)	32	89	22	92	112	65	32	89
6S B.s.(6S E.c.)	(7)	36	90	48	89	116	69	196	46
18S D.m.(18S H.s.)	4v6w	492	84	1566	52	802	74	822	76
18S H.s.(18S D.m.)	4v6x	406	86	1474	48	686	78	722	76
18S H.s.(18S S.c.)	4v6x	402	87	1474	48	746	76	768	76
18S S.c.(18S H.s.)	4v7r	434	85	1408	50	670	76	668	76
18S D.m.(18S S.c.)	4v6w	548	81	1566	52	824	74	860	75

18S S.c.(18S D.m.)	4v7r	414	85	1408	50	684	75	676	77
tRNA Gly G.k.(tRNA Gly H.s.)	4mgm	8	95	10	92	12	92	12	92
tRNA Gly H.s.(tRNA Gly G.k.)	5E6M	4	97	24	79	8	95	8	95
SRP H.s.(SRP C.l.)	4p3e	26	95	264	54	50	90	50	90
SRP C.l.(SRP H.s.)	4ue5	26	95	296	52	40	93	40	93
IRES HCV H.s.(IRES HCV O.c.)	5a2q	24	95	224	48	36	93	36	93
IRES HCV O.c.(IRES HCV H.s.)	4UJC	24	95	116	71	52	90	52	90
SAM B.s.(SAM C.s.)	4kqy	24	88	20	90	120	48	20	90
SAM B.s.(SAM T.t.)	4kqy	20	90	20	90	100	56	20	90
SAM C.s.(SAM B.s.)	5fkh	12	94	14	91	50	71	14	91
SAM C.s.(SAM T.t.)	5fkh	0	100	14	91	12	94	12	94
SAM T.t.(SAM C.s.)	3gx3	0	100	16	91	16	91	16	91
SAM T.t.(SAM B.s.)	3gx3	8	96	16	91	52	71	16	91
5S S.c.(5S E.c.)	5gak	26	87	44	75	58	70	22	85
5S E.c.(5S S.c.)	1C2X	38	80	78	43	42	75	40	78
5S S.c.(5S T.t.)	5gak	12	92	44	75	64	70	36	85
5S S.c.(5S T.c.)	5hd1	24	85	46	73	36	85	26	85
5S T.c.(5S S.c.)	5gak	30	87	44	75	46	79	36	85
5S T.t.(5S S.c.)	5t5h	36	82	88	43	44	81	40	82
5S H.m.(5S T.t.)	1ffk	20	92	22	89	32	84	16	90
5S T.t.(5S H.m.)	5hd1	20	88	46	73	28	88	24	87
5S S.c.(5S E.c.)	5gak	26	87	44	75	60	72	34	85
5S E.c.(5S S.c.)	5l3p	20	87	68	40	32	85	24	83
5S E.c.(5S T.c.)	5l3p	38	75	68	40	50	72	36	85
5S T.c.(5S E.c.)	5t5h	34	81	88	43	76	51	40	83
5S E.c.(5S T.t.)	5l3p	16	93	68	40	20	90	16	92
5S T.t.(5S E.c.)	5hd1	26	88	46	73	26	88	24	90
RNaseP a synt.(RNaseP a T.t.)	2a2e	96	84	158	77	106	83	106	83
RNaseP a T.t.(RNaseP a synt.)	3q1r	98	82	162	76	144	77	144	77
cobalamin mar.met.(cobalamin synt.)	4GXY	68	77	116	70	104	72	180	48
cobalamin synt.(cobalamin mar.met.)	4gma	56	80	56	80	96	65	40	88
lysine synt.(lysine T.m.)	3diz	36	87	22	93	40	89	8	98
lysine T.m.(lysine synt.)	4erl	8	98	32	88	12	96	12	96
glmS C.s.(glmS synt.)*	3b4c	28	87	56	78	92	46	56	78
glmS synt.(glmS C.s.)*	3l3c	56	76	68	74	92	59	64	77
glmS C.s.(glmS synt.)*	3b4c	4	98	88	56	96	55	76	70
glmS synt.(glmS C.s.)*	3l3c	56	80	100	59	102	54	76	71
glmS C.s.(glmS synt.)*	3b4c	14	94	76	65	82	62	48	81
glmS synt.(glmS C.s.)*	3l3c	64	77	88	63	78	70	52	82
16S E.c.(16S T.t.)	4v4q	166	93	908	64	492	82	280	90
16S T.t.(16S E.c.)	2ZM6	152	94	972	67	388	86	262	91
16S E.c.(16S P.f.)	4v4q	420	84	908	64	492	80	310	89
16S T.t.(16S P.f.)	2ZM6	418	83	972	67	490	81	254	91
16S P.f.(16S T.t.)	4v6u	412	84	918	65	624	77	512	81
16S P.f.(16S E.c.)	4v6u	412	82	918	65	516	79	522	81

*Three structural versions were obtained by removing pseudoknots.

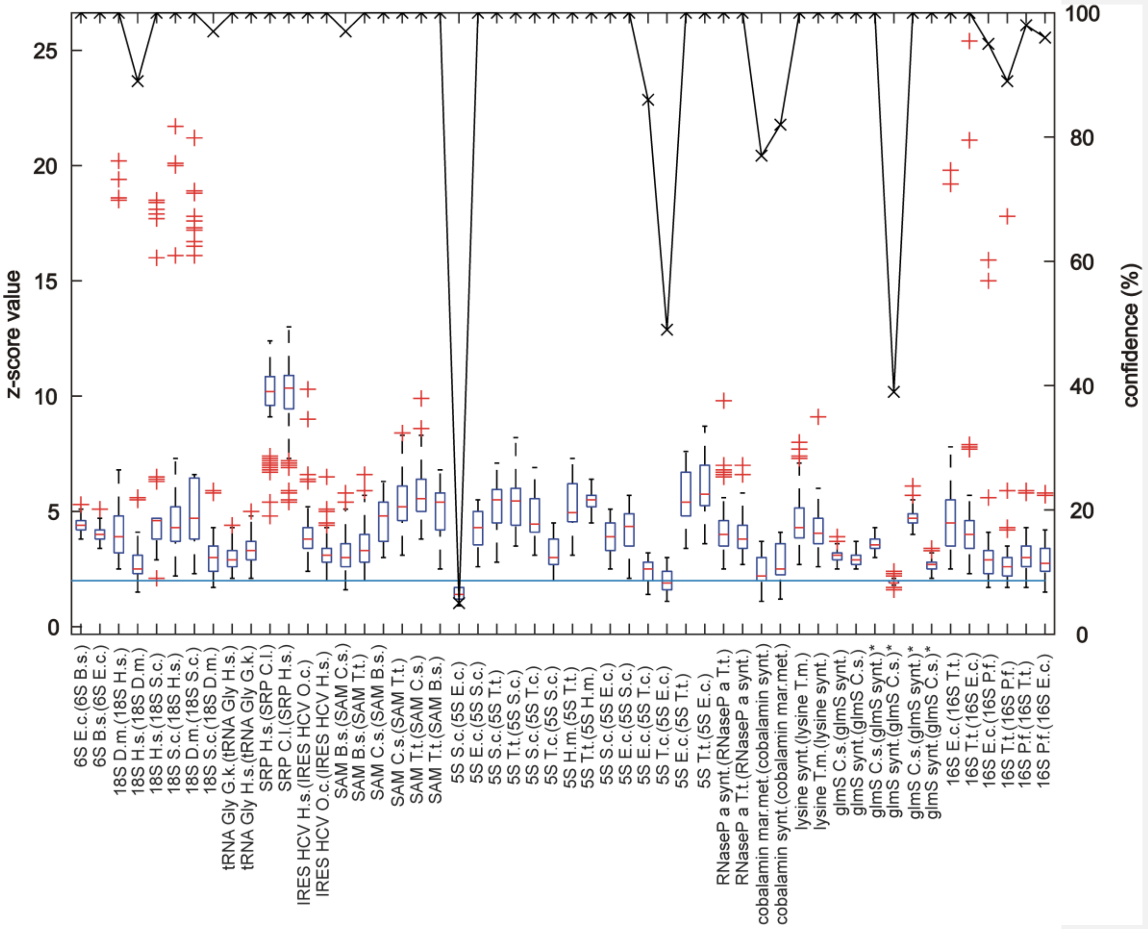

Figure S1. Variance of tree edit distance-based z-scores. z-scores were computed by repeating the bootstrap 100 times (100 runs with 100 randomized sequences each) for the 52 generated structures of the cross-validation dataset. X-axis shows generated structures. The left y-axis shows values of z-scores and their characteristics shown in a form of boxplot. The right y-axis shown percentage of the z-scores less than 2 shown as black line with crosses. The horizontal blue line shows the limit of reliability at z_d=2.

Table S5. Large-scale evaluation. The predicted/generated structures were evaluated by edit tree distances (computed by RNAdistance) to the templates. Mean of the distances for RNA families is shown. Note, that the lesser the value, the lesser the distance and the higher the similarity. The values depend on the size of compared structures.

RNA	Proposed method	RNAfold	refold method	Rsearch-based method
u4	39.8	96.6	86.9	82.8
tRNA Gly	8.90	24.9	20	20
oxyS	21.1	27.5	31.3	25.6
u5	22.8	38.3	46.1	29.9
FMN riboswitch	36.1	60.4	50.4	46
THF riboswitch	31.4	43.2	45.2	30.7
SRP bact small	36	36	45.8	30.2
SRP Metazoa	73.9	224.6	95.4	95.2
TPP riboswitch	8.50	47.1	44	44
IRES HCV	137.4	206.2	181.6	168
SAM_I_Tt_2GIS	35.5	45.1	58.4	39.9
Lysine riboswitch	72.5	85	132.3	87.2

Formatted Table

6S	67.2	81.6	120.1	73.1
spot42	14.4	50.8	40.9	36
gcvB	46.2	110.7	101.9	94.9
Bs2	107.8	121.9	115.1	113.9
ryhB	22.6	57	35.4	33
ms1	77.7	126.2	104	94.4
Msmest	125	176.2	170.1	137.5
TR	114.9	266.8	216	209.3
u2	31.7	101.8	87.4	80
u1	29.4	71.6	56.3	42.1
5S	5.50	65.6	45.1	44.3
RNaseP nuc	134.7	231.1	198.4	169.5
CPEB3 ribozyme	4.50	46.3	42.2	42.3
RNaseP arch	189.8	241.4	250.6	195.1
Cobalamin riboswitch	94.2	131.1	115.7	106.7
u6	22.9	89.3	81.4	80.2
Tetrahymena ribozyme	218.8	269.2	289.9	255.2
group I ribozyme	221.5	229.3	253.2	227.3
Mammalian 18S	166.9	1521.3	800.8	801.1
RNaseP bact a	104.9	215.6	192.3	133.4

Table S6. Tree edit distances of predicted/generated structures of gcvB homologs to the experimentally identified gcvB structure. Red color indicates an example of non-homologous RNA that should be distinguished from the gcvB homologs by its predicted/generated structure as a true negative. The example was the *E. coli* gcvB sequence with randomly shuffled dinucleotides. The sequence of the *E. coli* gcvB sequence with randomly shuffled dinucleotides and its structures predicted/generated by the compared methods are included in supplementary file S4.fasta.

	E. coli gcvB	*M. succiniciproducens* gcvB	*H. ducreyi* gcvB	*V. cholera* gcvB	Shuffled *E. coli* gcvB
Presented method	3	59	71	68	145
CentroidHomfold	84	96	138	123	- **
LocaRNA*	-	-	-	-	-
RNAfold	89	95	147	108	209
refold method	79	133	133	140	151
Turbofold	59	93	109	104	137

* LocaRNA's consensus structures can not be compared with RNAdistance as it may contain non-sequence characters.

**CentroidHomfold did not predict the structures of randomized *E.coli* gcvB sequence for an unknown reason.

Table S7. Tree edit distances of predicted/generated 18S rRNA structures to experimentally identified template of *H. sapiens* 18S rRNA.

	Galeopterus variegatus 18S	*Sarcophilus harrisii* 18S	*Sus scrofa* 18S
Presented method	106	441	697
CentroidHomfold			
LocaRNA*	-	-	-
RNAfold	1494	1599	1767
refold method	820	1095	1125
Turbofold	1312	1391	1649

* LocaRNA's consensus structures can not be compared with RNAdistance.

Table S8. List of supplementary fasta files.

File name	Description

S1.fasta	Sequences and structures of experimentally identified RNAs for cross-validation. Secondary structures were extracted from PDB structures using RNApdbee (17). The PDB structures are listed in Table S4.
S2.fasta	Sequences and structures for the large-scale evaluation dataset.
S2a.fasta	Templates for the large-scale evaluation dataset.
S3.fasta	Sequences and structure for gcvB example. In a), the sequence and structure of the *S. typhimurium* gcvB RNA with experimentally identified structure. In b) sequences of gcvB homologs and their structures predicted by available methods. Note that Locarna's consensus structures can be longer than the corresponding sequences. Turbofold input were the sequences of the homologs.
S4.fasta	Sequence and structures of *E. coli* gcvB with randomly shuffled dinucleotides.
S5.fasta	Sequences and structure for 18S rRNA example. In a), sequence and structure of *H. sapiens* 18S rRNA. In b), sequences of 18S rRNA homologs with their structures predicted by the compared methods. Note that Locarna's consensus structures can be longer than the corresponding sequences. Turbofold input were the sequences of the homologs.

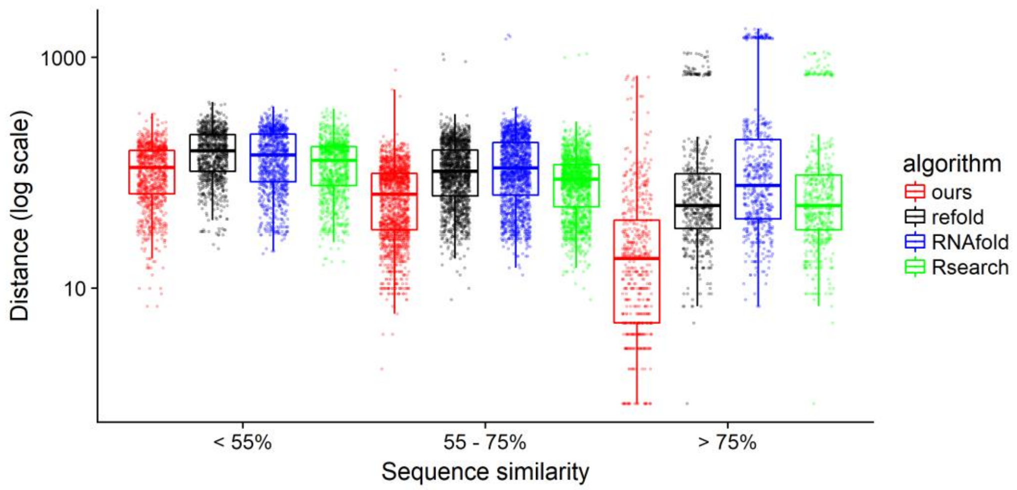

Figure S2. Comparison of the presented method with respect to sequence similarity. The comparison was carried out to demonstrate that the higher accuracy of the presented method indicated by Figure 4 was not made by several high-scoring sequences in the families. The compared methods were RNAfold as a representative of classical, single sequence secondary RNA structure prediction, a refold.pl-based method and Rsearch-based method that both allow for the principally same type of prediction as the presented method. Sequences of the large scale dataset (described in Tables S2 and S3) were used. In the figure, the sequences were sorted according to their sequence identity to templates to three classes as indicated by x-axis. Each class is evaluated with four box plots for the presented method (red), RNAfold (black) and the Rsearch-based method (green). Y-axis shows tree edit distances of generated/predicted structures to the templates (log scale).

REFERENCES

L. Argaman and S. Altuvia: fhlA repression by OxyS RNA: kissing complex formation at two sites results in a stable antisense-target RNA complex. *J Mol Biol*, 300(5), 1101-12 (2000)
C. L. Will and R. Luhrmann: Spliceosomal UsnRNP biogenesis, structure and function. *Curr Opin Cell Biol*, 13(3), 290-301 (2001) doi:S0955-0674(00)00211-8 [pii]
C. L. Will and R. Luhrmann: Spliceosome structure and function. *Cold Spring Harb Perspect Biol*, 3(7) (2011) doi:cshperspect.a003707 [pii]
10.1101/cshperspect.a003707
K. M. Wassarman and G. Storz: 6S RNA regulates E. coli RNA polymerase activity. *Cell*, 101(6), 613-23 (2000)
T. Moller, T. Franch, C. Udesen, K. Gerdes and P. Valentin-Hansen: Spot 42 RNA mediates discoordinate expression of the E. coli galactose operon. *Genes Dev*, 16(13), 1696-706 (2002)
C. M. Sharma, F. Darfeuille, T. H. Plantinga and J. Vogel: A small RNA regulates multiple ABC transporter mRNAs by targeting C/A-rich elements inside and upstream of ribosome-binding sites. *Genes Dev*, 21(21), 2804-17 (2007) doi:21/21/2804 [pii]
10.1101/gad.447207
Y. Ando, S. Asari, S. Suzuma, K. Yamane and K. Nakamura: Expression of a small RNA, BS203 RNA, from the yocI-yocJ intergenic region of Bacillus subtilis genome. *FEMS Microbiol Lett*, 207(1), 29-33 (2002) doi:S0378109701005511 [pii]
B. M. Davis, M. Quinones, J. Pratt, Y. Ding and M. K. Waldor: Characterization of the small untranslated RNA RyhB and its regulon in Vibrio cholerae. *J Bacteriol*, 187(12), 4005-14 (2005)
J. Panek, L. Krasny, J. Bobek, E. Jezkova, J. Korelusova and J. Vohradsky: The suboptimal structures find the optimal RNAs: homology search for bacterial non-coding RNAs using suboptimal RNA structures. *Nucleic Acids Res*, 39(8), 3418-26 (2011) doi:gkq1186 [pii]
10.1093/nar/gkq1186
J. Hnilicova, J. Jirat Matejckova, M. Sikova, J. Pospisil, P. Halada, J. Panek and L. Krasny: Ms1, a novel sRNA interacting with the RNA polymerase core in mycobacteria. *Nucleic Acids Res*, 42(18), 11763-76 (2014) doi:gku793 [pii]
10.1093/nar/gku793
S. D. Bentley, K. F. Chater, A. M. Cerdeno-Tarraga, G. L. Challis, N. R. Thomson, K. D. James, D. E. Harris, M. A. Quail, H. Kieser, D. Harper, A. Bateman, S. Brown, G. Chandra, C. W. Chen, M. Collins, A. Cronin, A. Fraser, A. Goble, J. Hidalgo, T. Hornsby, S. Howarth, C. H. Huang, T. Kieser, L. Larke, L. Murphy, K. Oliver, S. O'Neil, E. Rabbinowitsch, M. A. Rajandream, K. Rutherford, S. Rutter, K. Seeger, D. Saunders, S. Sharp, R. Squares, S. Squares, K. Taylor, T. Warren, A. Wietzorrek, J. Woodward, B. G. Barrell, J. Parkhill and D. A. Hopwood: Complete genome sequence of the model actinomycete Streptomyces coelicolor A3(2). *Nature*, 417(6885), 141-7 (2002)
K. Nagai, Y. Muto, D. A. Pomeranz Krummel, C. Kambach, T. Ignjatovic, S. Walke and A. Kuglstatter: Structure and assembly of the spliceosomal snRNPs. Novartis Medal Lecture. *Biochem Soc Trans*, 29(Pt 2), 15-26 (2001)
S. M. Marquez, J. K. Harris, S. T. Kelley, J. W. Brown, S. C. Dawson, E. C. Roberts and N. R. Pace: Structural implications of novel diversity in eucaryal RNase P RNA. *RNA*, 11(5), 739-51 (2005) doi:rna.7211705 [pii]
10.1261/rna.7211705
K. Salehi-Ashtiani, A. Luptak, A. Litovchick and J. W. Szostak: A genomewide search for ribozymes reveals an HDV-like sequence in the human CPEB3 gene. *Science*, 313(5794), 1788-92 (2006) doi:313/5794/1788 [pii]
10.1126/science.1129308
M. Skilandat, M. Rowinska-Zyrek and R. K. Sigel: Solution structure and metal ion binding sites of the human CPEB3 ribozyme's P4 domain. *J Biol Inorg Chem*, 19(6), 903-12 doi:10.1007/s00775-014-1125-6

C. Quast, E. Pruesse, P. Yilmaz, J. Gerken, T. Schweer, P. Yarza, J. Peplies and F. O. Glockner: The SILVA ribosomal RNA gene database project: improved data processing and web-based tools. *Nucleic Acids Res*, 41(Database issue), D590-6 (2012) doi:gks1219 [pii]
10.1093/nar/gks1219
M. Antczak, T. Zok, M. Popenda, P. Lukasiak, R. W. Adamiak, J. Blazewicz and M. Szachniuk: RNApdbee--a webserver to derive secondary structures from pdb files of knotted and unknotted RNAs. *Nucleic Acids Res*, 42(Web Server issue), W368-72 doi:gku330 [pii]
10.1093/nar/gku330
